# Grid-like Processing of Imagined Navigation

**DOI:** 10.1016/j.cub.2016.01.042

**Published:** 2016-03-21

**Authors:** Aidan J. Horner, James A. Bisby, Ewa Zotow, Daniel Bush, Neil Burgess

**Affiliations:** 1 UCL Institute of Cognitive Neuroscience, 17 Queen Square, London WC1N 3AZ, UK; 2 UCL Institute of Neurology, Queen Square, London WC1 3BG, UK

## Abstract

Grid cells in the entorhinal cortex (EC) of rodents [1] and humans [2] fire in a hexagonally distributed spatially periodic manner. In concert with other spatial cells in the medial temporal lobe (MTL) [3, 4, 5, 6], they provide a representation of our location within an environment [7, 8] and are specifically thought to allow the represented location to be updated by self-motion [9]. Grid-like signals have been seen throughout the autobiographical memory system [10], suggesting a much more general role in memory [11, 12]. Grid cells may allow us to move our viewpoint in imagination [13], a useful function for goal-directed navigation and planning [12, 14, 15, 16], and episodic future thinking more generally [17, 18]. We used fMRI to provide evidence for similar grid-like signals in human entorhinal cortex during both virtual navigation and imagined navigation of the same paths. We show that this signal is present in periods of active navigation and imagination, with a similar orientation in both and with the specifically 6-fold rotational symmetry characteristic of grid cell firing. We therefore provide the first evidence suggesting that grid cells are utilized during movement of viewpoint within imagery, potentially underpinning our more general ability to mentally traverse possible routes in the service of planning and episodic future thinking.

## Highlights

• Grid cells fire in a spatial pattern as mammals actively navigate their environment
• Grid cells might move the viewpoint of imagination, aiding goal-directed navigation
• Human entorhinal cortex shows a grid-like fMRI signal during imagined navigation
• This signal is similar to that seen in the same people during virtual navigation

## Teaser

Horner et al. find a grid-like fMRI signal in human entorhinal cortex as participants imagine navigating through space, similar to that seen during navigation in virtual reality. Thus, grid cells may move the viewpoint of imagination, extending their suggested role in updating self-location during navigation to include dynamic imagery and planning.

## Results and Discussion

We searched for an fMRI signal in human entorhinal cortex (EC) consistent with the presence of grid cell activity during imagined navigation. Grid cell firing patterns have a consistent orientation [19, 20], and this macroscopic organization can be observed with fMRI when participants navigate a virtual reality (VR) environment [10, 21]. This grid-like signal reflects a difference in blood-oxygen-level-dependent (BOLD) activity in EC when participants are moving along one of the six grid axes (“on-axis”) versus between them (“off-axis”). Critically, this signal is seen during periods of virtual movement compared to stationary periods and has specifically 6-fold rotational symmetry (or 60° periodicity) as a function of movement direction [10]. Importantly, a generalization of the role of grid cells in virtual navigation to imagined movement of viewpoint would imply the same grid orientation during virtual and imagined navigation in the same environment.

**Figure 1:**
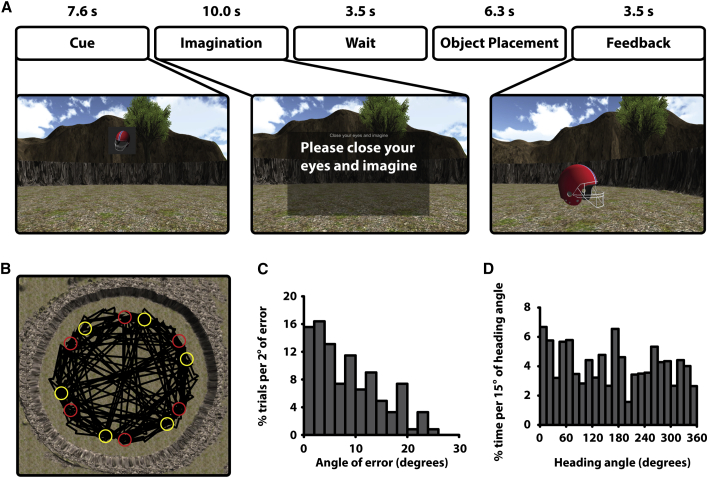
Experimental Design and Behavioral Data (A) Trial structure during the imagination block, showing example screen shots for the cue, imagination, and feedback periods. Participants were cued with a single object at the top of the screen and required to rotate such that they were facing toward the remembered location of that object. They then closed their eyes and imagined moving from their current location to the remembered location of the object. Following this, they waited for a jittered period of time (2–6 s) before moving to the object location and pressing a button. The object then appeared in the correct location, and participants had to navigate to it during the feedback period prior to the start of the next trial. The timing for each period was user defined by either pressing a button (during cue, imagination, and object placement) or moving into the object (during feedback). Times (in s) above each period label show the mean time across all trials and participants for each period. (B) A bird’s-eye view of the circular arena with an example path across both imagination blocks for a single participant in black and object locations for the two blocks in red and yellow, respectively. (C) Histogram showing the percentage of trials per 2° of heading angle error for the object placement task for a single participant. (D) Histogram showing percentage of time across both imagination blocks per 15° of heading angle for a single participant. (B–D) Data shown are from the participant with the median heading angle error across all participants (see Figure S1 for data across all participants).

A VR object-location memory task was used during fMRI scanning. After learning six object locations, participants were required to both move to and imagine moving to the locations of each object during a period that included both a retrieval and imagination element (henceforth referred to as the imagination block) (Figure 1; see Experimental Procedures). Participants navigated (and imagined navigating) every possible path between the six objects in both directions twice during each block (6 objects, 30 paths, 60 trials per block, 2 blocks per participant). Locations of objects, and therefore paths between objects, were chosen to ensure full coverage of heading directions (sampling every 15°) in the full 0–360° range. During this period, we defined “movement,” “stationary,” and “imagination” periods and interrogated the data for a grid-like signal during each period.

**Figure 2:**
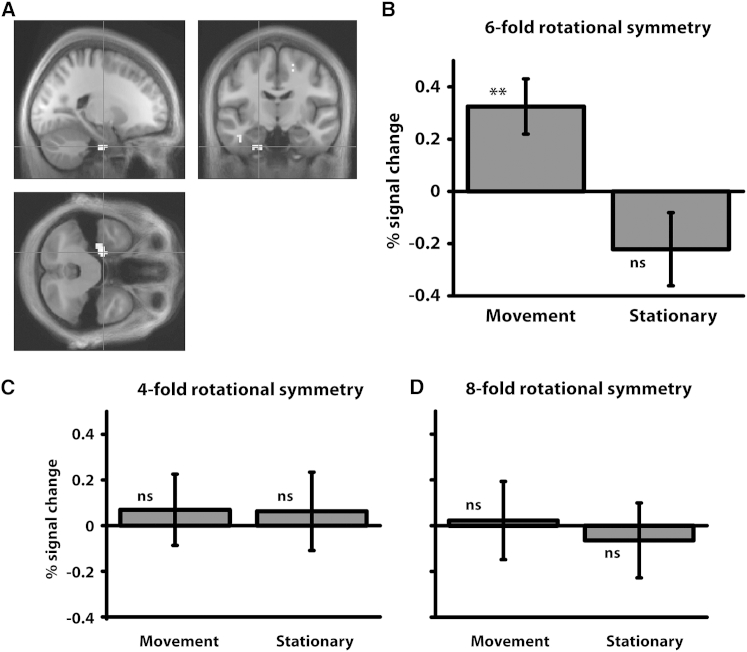
Movement-Related Grid-like Signal (A) Sinusoidal modulation of BOLD response by heading angle with 6-fold rotational symmetry for movement > stationary periods in EC (−21, −12, −36; p < .05 SVC; shown at p < .005 unmasked for display purposes; see Figure S3B for a masked image), from the split-half analysis where grid orientation was estimated on half the data and applied to the other half, separately for movement, stationary, and imagination periods. (B) % signal change from peak shown in (A) for 6-fold rotational symmetry during movement and stationary periods. (C and D) % signal change for peak shown in (A) for 4- and 8-fold rotational symmetries during movement and stationary periods. Note that we saw no effect for 4- or 8-fold symmetries in the entire EC, i.e., the null effect shown here is not specific to the region of interest based on the 6-fold analysis. Error bars show ±1 SE; ^∗∗^p < .01; ns, not significant (relative to baseline).

Participants performed the object-location task accurately, with a median angular error of 7.36° and a median distance error of 15.86 virtual meters (vm; radius of circular arena: 55 vm; Figures 1 and S1). We first sought evidence for grid-like activity in EC during movement periods. We split the data into halves, calculating the orientation of a 60° periodic signal in one half of the data and looking for evidence for that grid orientation in the second half. This process was performed separately for movement, stationary, and imagination periods (see Experimental Procedures). Restricting our search to EC, we looked for a greater 6-fold signal during movement than stationary periods. This revealed a significant cluster in left EC (−21, −12, −36; p < .05 small volume corrected [SVC] in a bilateral EC volume, see Experimental Procedures; see Figures 2A and S3B for overlap with EC). An additional cluster in right EC that failed to survive SVC (+24, −15, −33; p < .005 uncorrected) was also seen. The peak EC voxel (defined by the movement > stationary effect) showed a significant 6-fold modulation during movement (relative to baseline of no parametric modulation; *t*(25) = 3.08, p < .01), but not stationary (*t*(25) = 1.58, p = 0.13), periods (Figure 2B).

This grid-like pattern (6-fold modulation during movement) did not correlate with behavioral accuracy (median angular error) across participants (R^2^ = 0.07, p = 0.19) nor was there any consistent grid orientation across participants (Rayleigh test, p’s > .05), suggesting behavioral performance or task structure cannot fully explain this pattern. The reverse contrast (stationary > movement) failed to reveal any significant clusters in EC. Thus, we see a movement-specific 6-fold symmetric pattern of activity in EC. Conducting the same analyses for other rotational symmetries, we found no evidence for a 4- or 8-fold symmetric signal in EC (movement > stationary; Figures 2C and 2D; see Figure S2B for 3-, 5-, and 7-fold symmetry). Thus, we could find no evidence for other movement-related rotational symmetries. This specifically 6-fold signal is consistent with the presence of a population of cells with a coherent 60° periodic modulation of activity by movement direction, grid cells in EC being the only cell type known to have this property.

**Figure 3:**
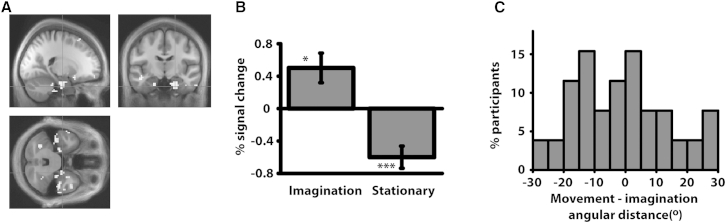
Imagination-Related Grid-like Signal (A) Sinusoidal modulation of BOLD response by heading angle with 6-fold rotational symmetry for imagination > stationary periods in EC (+21, −12, −33; p < .05 SVC; shown at p < .005 unmasked for display purposes; see Figure S3C for a masked image), from the analysis where grid orientation was estimated during all movement periods and applied to the imagination and stationary periods. (B) % signal change from peak shown in (A) for 6-fold rotational symmetry during imagination and stationary periods. Error bars show ±1 SE; ^∗∗∗^p < .001; ^∗^p < .05 (relative to baseline). (C) Histogram showing the percentage of participants per 5° of angular distance between grid orientations during movement and imagination periods (circular mean across participants = −5.5°).

Addressing the critical question of whether similar grid-like processing occurs during imagined navigation periods, we estimated the grid orientation during all movement periods and searched for evidence of grids with this orientation during both stationary and imagination periods (see Experimental Procedures). We contrasted imagination versus stationary periods (similar to the above movement > stationary analysis), revealing significant clusters in both right (+21, −12, −33) and left (−21, −15, −30) EC (p < .05 SVC; Figures 3A and S3C for overlap with EC). There was also a significant effect for imagination > baseline (i.e., irrespective of stationary periods) in right EC (+15, −9, −27, p < .05 SVC). The peak voxel (defined by the imagination > stationary effect, in right EC) showed a significant positive effect during imagination periods (relative to baseline; *t*(25) = 2.75, p < .05; Figure 3B), providing evidence for a grid-like signal during imagination periods with similar orientation as during movement. The mean angular difference between movement and imagination grid orientations was −5.5° (Figure 3C; NB the distribution of differences was not significantly clustered; Rayleigh test p = 0.12).

The peak imagination > stationary voxel also showed an unexpected negative effect in the stationary period (relative to baseline; *t*(25) = 4.39, p < .001; Figure 3B), implying periodic modulation opposite to that during movement. Such an effect could either be due to adaptation, whereby previously active cells during movement or imagination periods show reduced firing, or a grid-like signal during stationary periods that is rotated 30° relative to movement periods. Given that no positive or negative grid-like signal was seen in the above split-half analysis (where orientation was estimated independently for the stationary period), it is not clear what this effect reflects, although a rebound from inhibition during surrounding periods of movement or imagination is possible given the importance of inhibition in EC [22, 23, 24]. Overall, we failed to find evidence for a consistent grid-like signal during stationary periods (and definitively not one aligned to that during navigation), in contrast to the signal seen during periods of imagined navigation.

Finally, we used the same split-half analysis employed above for movement versus stationary periods (where orientation was estimated separately for each period) to further investigate imagination versus stationary periods. This analysis revealed a 6-fold symmetric signal in right EC (+18, −21, −21; p < .001 uncorrected; Figure S2C), though we note this effect was marginal at the SVC level (p = 0.07 SVC). As in the movement versus stationary analyses, no EC effects were found for either a 4-fold or 8-fold symmetric signal (imagination > stationary; Figure S2D for 3- to 8-fold analyses).

Grid cells in EC are thought to be recruited during spatial imagery, supporting goal-directed navigation. Despite this hypothesis, no direct evidence has been provided for grid cell activity during non-movement periods when participants are engaged in imagining future navigation. Using fMRI, we provide evidence suggesting the presence of a grid-like signal in human EC during periods of imagined navigation when participants are not actively moving. Critically, we demonstrate that a 6-fold fMRI pattern is seen during imagined navigation, with a similar grid orientation to movement periods, and this pattern is not present during other periods of the task. These results complement recent fMRI findings showing heading direction signals in the human medial temporal lobe (MTL) [25, 26] that are utilized in a goal-directed manner [27] and for a general role of the MTL in goal-directed navigation [28, 29, 30, 31, 32]. We therefore provide the first evidence consistent with the hypothesis that grid cells are utilized during imagined navigation, allowing us to mentally traverse space and memory [11, 12, 13, 33, 34] in the service of planning and episodic future thinking [18].

## Experimental Procedures

### Participants

26 participants (9 female) were recruited through the UCL Institute of Cognitive Neuroscience subject panel. Participants gave informed consent, were reimbursed (£25), had a mean age of 23.7 (SD = 4.2), were right-handed, and were free from neurological impairment. The experiment was approved by the UCL Research Ethics Committee (1825/003).

### VR Environment

The environment was created using Unity (https://unity3d.com/): an empty circular arena with distal cues (mountains, trees, and buildings) beyond the circular wall to provide orientation information. Participants learned a series of object locations within this environment over the course of two blocks. Although new objects were introduced in the second block, the VR environment remained constant. Participants were lying in an MRI scanner and viewed the VR environment (projected on a screen behind their heads) via a mirror. A keypad was used to navigate, with buttons for turning left, turning right, and moving forward.

### Procedure

Before scanning, participants practiced the task, learning a series of object locations and practicing the retrieval and imagination task until the experimenter was confident that they were able to navigate accurately and understood the imagination task (taking 5–15 min).

During scanning, participants completed two encoding and imagination blocks. During encoding, six object locations were learned. A single object was presented within the arena, and participants navigated to the object to trigger the end of the trial, when the object disappeared and another object appeared in a different location. Each object was seen five times (in the same location).

Following encoding, participants were required to remember object locations and imagine moving to the correct location. At the start of each trial, an object was presented at the top of the screen. During this period, participants’ viewpoint could rotate but not move forward or backward. They were required to orient themselves toward the remembered location of the cued object. Once oriented, they pressed a keypad button and an instruction appeared to “close your eyes and imagine.” During this period, they closed their eyes and imagined moving from their current location to the remembered location of the object as directly as possible (i.e., in a straight line along the direction they had chosen). They were told to imagine moving through the environment as vividly as possible at a similar pace to their actual movement within the environment. Once they had completed this imagined navigation, they pressed a button and opened their eyes. An instruction appeared to “wait to move” for a jittered wait period lasting 2–6 s (randomly selected on each trial) during which participants could not move or rotate their viewpoint. Following this, participants were required to move toward the remembered object location as directly as possible (similar to the imagination phase). Once in the remembered location, they pressed a button and the location was recorded. The object then appeared in the correct location, and participants navigated to it before the next trial started.

In each imagination block, every path between each of the six objects was navigated and imagined twice, resulting in 60 trials. Following the first encoding and imagination block, a second set of object locations was encoded and tested. The locations for each object across the two blocks were chosen such that all 24 heading directions within the 360° range were sampled at a resolution of 15° (Figures 1B and 1D). Three trial orders in the encoding and imagination blocks were created (each pseudo-randomly generated) and counterbalanced across participants.

### fMRI Acquisition

48 T2^∗^-weighted slices (64 × 74, 3 mm × 3 mm, TR = 70 ms, TE = 30 ms, repetition time = 3,360 ms) per volume were acquired using echo-planar imaging (EPI) on a 3T Trio system (Siemens) with a 32-channel head coil. Slices were tilted 45° up at the front and acquired in ascending order. The number of volumes during each imagination block varied, with a mean of 525 (range: 346–707). The first five volumes of each session were discarded to allow for T1 equilibrium. A double-echo FLASH field-map for distortion correction of the EPI volumes was acquired, as well as a three-dimensional MDEFT structural image (1 mm^3^) for normalization to the MNI template.

### fMRI Analyses

We only analyzed data from the two imagination blocks. EPI images were bias corrected, unwarped, realigned, slice time corrected, normalized, and smoothed (8 mm FWHM) using SPM8 (http://www.fil.ion.ucl.ac.uk/spm/).

General linear models (GLMs) were constructed in MATLAB and SPM8. We first performed a split-half analysis where the grid orientation for movement, stationary, and imagination periods were estimated independently. In a first-level GLM, we split the data into interleaved 30 s time bins. Orientation was estimated for odd-numbered bins and then subsequently applied to the even-numbered bins. Any translational movement >2 s was defined as “movement,” and any period of >2 s where no translational movement occurred (excluding the imagination period) was defined as “stationary.” The imagination period was defined by the participant button presses at the start and end of the cued imagination period (remaining time was unmodeled). Six regressors modeling movement, stationary, and imagination periods in the two halves of the data were created for each imagination block.

In the first analysis, two parametric modulators (PMs) were created for the movement, stationary, and imagination periods for the first half of the data per block (i.e., the odd-numbered time bins; no PMs were applied to the second half of the data) and entered into a GLM. For the main analysis, looking for 60° periodicity in dependence on heading direction, the two PMs were cos(6θ(t)) and sin(6θ(t)), where θ(t) is the heading angle at time t. The weights (or “betas,” b1 and b2) of these cosine and sine regressors in the GLM fitted to the fMRI time series were found for voxels within an anatomically defined bilateral EC (Figure S3A) region of interest. We then calculated the orientation of periodic dependence on direction separately for the movement, stationary, and imagination periods, using the mean values of these weights (< b1 > and < b2 >), as Ф = [arctan(< b2 > /< b1 >)]/6 (separately for each block). This uses the cosine and sine regressors as a quadrature filter to detect the angle of any variation in fMRI signal with heading direction that has 60° periodicity: e.g., if < b1 > is large and < b2 > is small, variation is aligned to 0°; if < b2 > is large and < b1 > is small, variation is aligned to 15°; see [10].

In a second analysis, we looked for 60° periodicity with these orientations in the second half of the data (i.e., the even-numbered time bins; no PMs were applied to the first half of the data) for movement, stationary, and imagination periods, respectively. Here, one PM was used for each of the second half movement, stationary, and imagination periods: a cosine of heading angle aligned to the orientation for that period, i.e., cos[6(θ(t)−Ф)]. The betas for these three regressors were analyzed across participants (“second-level” analyses). Each beta reflects the extent of 6-fold periodicity in variation of fMRI signal with direction during the corresponding periods. We made comparisons of the sizes of betas for movement > stationary and imagination > stationary periods (averaged across blocks). This analysis was repeated for the main comparisons of 4- and 8-fold rotational symmetries (as in [10]), as well as 3-, 5-, and 7-fold symmetries for completeness. Comparisons of single conditions relative to “baseline” refer to comparisons of betas for a single PM relative to the null hypothesis of no parametric modulation (a one-sample t test relative to zero).

Next, we asked whether there was periodic variation in fMRI signal during imagination and stationary periods with a similar orientation to that found during movement periods. Here, we modeled all movement, stationary, and imagination in a block with three separate regressors (i.e., not split-half). For each period, we included two further PMs, the cosine and sine of the heading angle, cos(6θ(t)) and sin(6θ(t)), and calculated the orientation of any 6-fold periodic variation, as above. In a second step, we used a single PM for each of the movement, stationary, and imagination periods to look for periodic variation aligned with the orientation found for the movement period, i.e., cos[6(θ(t)−Ф)], where Φ was the movement period orientation. Significantly positive betas for this regressor during stationary or imagination periods reflects the presence of 6-fold periodic dependence on heading direction with the same orientation as during movement.

Given our highly specific hypotheses regarding grid cells in EC, we report significant voxels corrected for multiple comparisons within an anatomically defined bilateral EC mask (Figure S3A), p < .05 SVC. For interest, we also report p < .005 uncorrected effects in EC; however, such effects should be treated with caution.

## Author Contributions

A.J.H., N.B., D.B., and J.A.B. designed the study. A.J.H. and J.A.B. collected the data. A.J.H., N.B., E.Z., and D.B. analyzed the data. A.J.H. and N.B. wrote the manuscript.

## Supplemental Information

Document S1. Figures S1–S3

Document S2. Article plus Supplemental Information
