# Supplementary material for: Grid-like Processing of Imagined Navigation
Source: Curr Biol. 2016 Mar 21;26(6):842–7. doi: 10.1016/j.cub.2016.01.042 (PMC4819517; doi:10.1016/j.cub.2016.01.042)
Supplement: Document S1. Figures S1–S3 [file mmc1.pdf]

Current Biology, Volume *26*

Supplemental Information

Grid-like Processing of Imagined Navigation

Aidan J. Horner, James A. Bisby, Ewa Zotow, Daniel Bush, and Neil Burgess

Supplemental Data

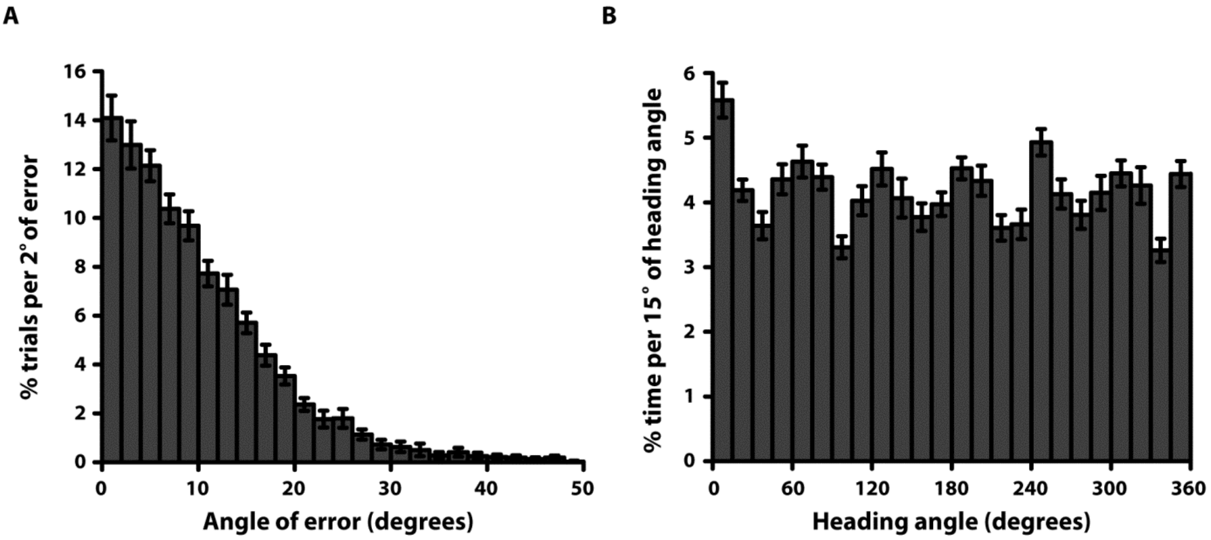

Figure S1, related to Figure 1. Mean angular error and heading angle. (A) Histogram showing the mean percentage of trials per 2° of heading angle error for the object placement task across participants and (B) histogram showing the mean percentage of time across both retrieval/imagination blocks per 15° of heading angle across participants. Error bars +/- 1 standard error.

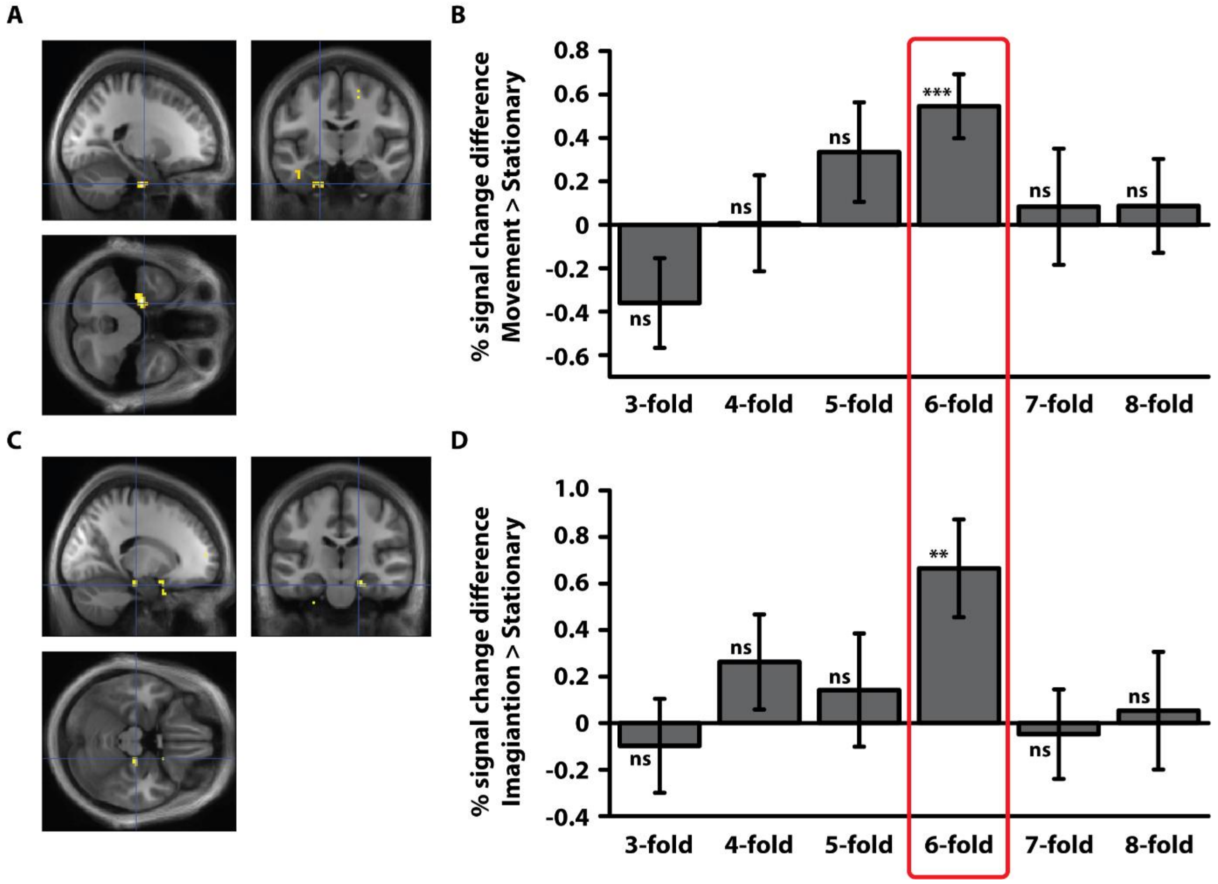

Figure S2, related to Figure 2. Movement and Imagination grid-like signals across rotational symmetries. (A) Sinusoidal modulation of BOLD response by heading angle with six-fold rotational symmetry for movement > stationary periods in entorhinal cortex (-21, -12, -36; p<.05 SVC; shown at p<.005 uncorrected; as shown in Figure 2A & S3B). (B) % signal change difference for peak shown in (A) between movement and stationary periods across 3-8 fold rotational symmetries. (C) Sinusoidal modulation of BOLD response by heading angle with six-fold rotational symmetry for imagination > stationary periods in entorhinal cortex (+18, -21, -21; shown at p<.005 unmasked for display purposes, see Figure S3D for a masked image). (D) % signal change difference for peak shown in (C) between imagination and stationary periods across 3-8 fold rotational symmetries. Error bars shown +/- 1 standard error; ***p<.001; **p<.01; ns = not significant (relative to baseline). All data from analyses that calculated independent orientations on half of the data and applied that orientation to the second half of the data (independently for movement, stationary and imagination periods).

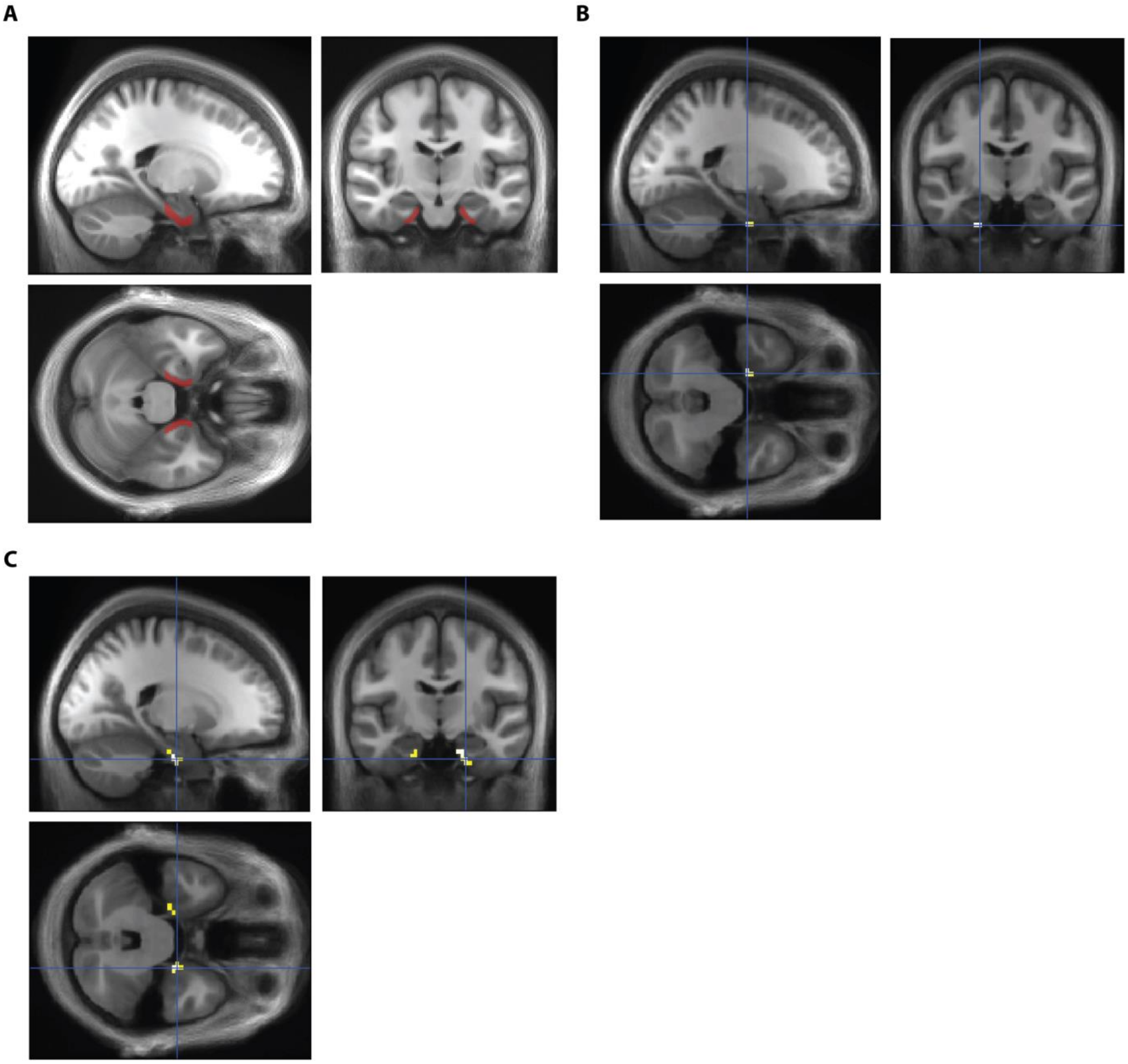

Figure S3, related to Figure 3. Entorhinal cortex mask and masked fMRI results. (A) Mean normalised structural image across participants with EC mask shown in red. (B) Masked movement > stationary from the split-half analysis, as shown in Figure 2A. (C) Masked imagination > stationary using the orientation taken from all movement periods, as shown in Figure 3A. Functional data in (B) & (C) masked using the EC template shown in (A) and thresholded at $p<.005$ uncorrected for display purposes.
